# Observations on Working Psychoanalytically with a Profoundly Amnesic Patient

**DOI:** 10.3389/fpsyg.2017.01418

**Published:** 2017-08-25

**Authors:** Paul A. Moore, Christian E. Salas, Suvi Dockree, Oliver H. Turnbull

**Affiliations:** ^1^Department of Psychiatry, School of Medicine, Trinity College, Dublin Dublin, Ireland; ^2^Friary Court Medical Centre Kilkenny, Ireland; ^3^Laboratory of Cognitive and Social Neuroscience, Faculty of Psychology, Diego Portales University Santiago, Chile; ^4^School of Psychology, Bangor University Bangor, United Kingdom; ^5^National Rehabilitation Hospital Dublin, Ireland

**Keywords:** amnesia, brain injury, emotion, memory, psychoanalysis, psychotherapy, transference

## Abstract

Individuals with profound amnesia are markedly impaired in explicitly recalling new *episodic* events, but appear to preserve the capacity to use information from other sources. Amongst these preserved capacities is the ability to form new memories of an *emotional* nature – a skill at the heart of developing and sustaining interpersonal relationships. The psychoanalytic study of individuals with profound amnesia might contribute to the understanding the importance of each memory system, including effects on key analytic processes such as transference and countertransference. However, psychoanalytic work in the presence of profound amnesia might also require important technical modifications. In the first report of its kind, we describe observations from a long term psychoanalytic process (72 sessions) with an individual (JL) who has profound amnesia after an anoxic episode. The nature of therapy was shaped by JL’s impairment in connecting elements that belong to distant (and even relatively close) moments in the therapeutic process. However, we were also able to document areas of preservation, in what appears to be a functioning therapeutic alliance. As regards transference, the relationship between JL and his analyst can be viewed as the evolution of a narcissistic transference, and case material is provided that maps this into three phases: (i) rejecting; (ii) starting to take in; and (iii) full use of the analytic space – where each phase exhibits differing degrees of permeability between JL and the analyst. This investigation appears to have important theoretical implications for psychoanalytic practice, and for psychotherapy in general – and not only with regard to brain injured populations. We especially note that it raises questions concerning the *mechanism* of therapeutic action in psychoanalysis and psychotherapy, and the apparent unimportance of episodic memory for many elements of therapeutic change.

“Why was he doubly irritated? Because he had forgotten and because he remembered that he had reminded himself twice not to forget”

James Joyce, *Ulysses*

## Introduction

Profound amnesia, after acquired brain damage, has long offered valuable insight into the neurological basis, and the neuropsychological mechanisms, of memory and learning (Milner et al., 1968; Schacter, 1992; Squire and Zola, 1997). The most important finding of this line of research has been the recognition of multiple independent memory systems. For example, individuals with profound amnesia –after hippocampal damage– are markedly impaired on *explicitly* recalling new episodic events, but appear to preserve the capacity to use information from other sources, such as procedural or skills-based memory, and critically when this information is of an emotional nature. Thus, clinical reports and behavioral studies suggest that individuals with profound amnesia are not only able to experience emotions (Feinstein et al., 2010; Damasio et al., 2012), but more importantly, can learn about the emotional valence of experiences and make decisions based upon that knowledge (Claparede, 1911/1951; Tranel and Damasio, 1990, 1993; Turnbull and Evans, 2006; Evans-Roberts and Turnbull, 2011). This preserved capacity is at the heart of developing and sustaining interpersonal relationships.

The existence of such separate memory systems is a topic that has increasingly attracted the interest of the psychoanalytic community. The main reason appears to be that such findings converge with recent psychoanalytic theorization regarding the role of explicit and implicit domains for psychic change (Clyman, 1991; Charles, 2005; Fosshage, 2005; Boston Change Process Study Group, 2008). In this context, the psychoanalytic study of individuals with profound amnesia might contribute to the understanding of the importance of each memory system, and its relevance for key psychoanalytic processes, such as transference and countertransference. To our knowledge, there is only one case report that has partially addressed this issue. Turnbull et al. (2006) described a psychoanalytic treatment (eight sessions) of an individual (Mr. N) who became deeply amnesic after an anoxic episode. The main goal of that case study was to explore whether Mr. N was able to learn from the dynamic interaction with the analyst, and to determine if such interaction modified the emotion related material during the sessions, despite his absence of episodic recall of the analyst, or even that the meetings had taken place. The authors observed that during the treatment, the emotional experience of Mr. N toward the analyst changed, showing ‘greater feelings of familiarity and levels of comfort.’ They concluded that such emotional change in the interaction may have reflected the ‘beginning of a transference relationship.’

Even though the case report of Mr. N offers valuable insight into the preservation of interpersonal emotional learning in amnesic patients, it nevertheless has important limitations. The most obvious is the short duration of the treatment (which lasted only 8 weeks) allowed only observation of the very *early* stages of the transferential process, and not a more complete examination of the transference phenomenon. Another theoretical limitation is that it focuses on the generation, and modification, of feelings from the patient toward the analyst (transference), not taking into account the analyst’s emotional experience toward the patient (countertransference). This is a limitation in two senses. Firstly, contemporary psychoanalytic views on transference and countertransference are regarded as two mutually influencing aspects of the *same* interpersonal process (Thomä and Kächele, 2010). Secondly, by not considering the countertransferential phenomenon, it does not take into account the impact of the patients’ disorganized mind on the analyst’s mind, and the ways in which the patient might *use* the analyst’s mind as a source of regulation (Pepping, 1993; Lewis, 1999; Coetzer, 2006; Salas, 2008; Klonoff, 2010; Salas et al., 2013).

The main goal of this article is to contribute a unique case to the existing literature on the neuropsychological basis of memory systems, and its potential relevance for psychoanalytic theory and technique. We describe observations from a *long term* psychoanalytic process (72 sessions) of an individual with profound amnesia after an anoxic episode. This case study addresses two main questions. Firstly, what is the impact of amnesia on transference and countertransference phenomena? Secondly, which technical and therapeutic modifications are required to work psychoanalytically with such amnesic individuals?

## Case Description

### Biographical Information

In the period of treatment, JL was a 38 years old single man.^1^ At the time of the brain injury, 3 years earlier, JL lived alone, at what was once the family home. He had been educated to Master’s degree level, and was employed as a professional. Three years before treatment, as a result of a complex medical condition related to diabetes, he had experienced an anoxic brain injury, and upon admission to hospital he suffered three cardiac arrests in quick succession. Consistent with the common neuropsychological consequences of such damage, JL then presented with a marked impairment in laying down new episodic memories (anterograde amnesia), but a largely preserved capacity to recall events *prior* to it (retrograde memory).

The rehabilitation service’s intake report for JL indicates that before the accident, there were significant premorbid systemic issues in his background. JL had two older brothers, two younger sisters and a younger brother. His life had been fractious and unsettled, with the family moving quite frequently for work – often internationally. There was one long distance move, where the family settled for approximately 10 years. On admission to the rehabilitation services, JL reported using and abusing alcohol from the age of thirteen. However, during treatment, he informed the starting date as even earlier (10 years of age).

JL was 20 years old when his parents separated, acrimoniously, and he recalls having to leave the country quickly, with his mother and siblings relocating to their country of origin, and living off the kindness and generosity of friends and family. JL met this adversity quite well, working during the day as an office clerk, and returning to education at night in order to obtain a Masters degree. He also trained for, and eventually worked in, a professional position. However, within a few years of returning (when JL was in his early twenties) both parents passed away. His father was the initial loss, followed 3 years later by his mother. JL spiraled into a full blown alcohol and cannabis addiction, which also led to deterioration in work performance, and subsequent loss of a series of work positions. At the same time JL began to neglect his personal well-being, his living arrangements, and more importantly his diabetes, which had been diagnosed when he was sixteen. This resulted in a series of heart attacks, and eventually the anoxic brain injury, as a result of a complex medical condition related to diabetes, a disorder often associated with mismanagement of insulin.

Since the injury, JL has lived in a residential unit for neurological patients. Prior to this he had spent a year in a rehabilitation hospital, which he appeared to have much preferred to the current accommodation. At the hospital his days were structured and interesting. At the residential unit he became the youngest service user, and did not have much in common with the other patients. He had (and has) an older brother who lives nearby, visiting JL once a week. However, JL had little contact with the rest of his family, who live abroad. At the residential unit, his routine was highly repetitive, with much of his day consisting of listening to music, doing jigsaws, and making models. There are further activities organized by the residential unit, and the rehabilitation services which he attended twice a week. Because of his diabetes he was monitored closely by the ‘sisters’ (as JL described them), in reality nurses. This lack of freedom was a great source of frustration, and JL expressed a strong desire to live independently, or to be moved to assisted housing.

### Clinical Presentation

At the initial consultation JL was dressed in jeans, and wearing a football jersey and training shoes – which were in need of cleaning. His hair was long and greasy, and came to just above the shoulder, around his neck hung a collection of odds and ends, suspended from what looked like a piece of climbing rope, attached to a large climbing clip. The objects were nail clippers, keys, pens, a miniature torch, a bottle opener, and a mobile phone. The latter JL used as a memory aid. He also wore glasses, attached to a cord around his neck. He had a day’s growth of beard, where large areas beneath the chin had been missed when last he shaved. His fingers were nicotine stained, and the nail on the little finger of his left hand had been allowed to grow much longer than its neighbors. This idiosyncrasy endured for the remainder of the therapy, a remarkably continuous feature despite JL’s amnesia.

Upon meeting JL for the first time, he presented as polite and friendly, and spoke both confidently and clearly, with a foreign accent. He appeared to be lucid and oriented for both time and space. When entering the consulting room he examined it with what seemed like an intelligent curiosity. However, after exchanging introductions, the amnesia presented itself almost immediately, with JL repeating information to the analyst within 5 min. The following vignette shows the impact of JL’s memory impairment in the continuity of the session, as well as the preservation of other abilities, such as his insight:

P (Paul Moore): Maybe I’ll take some notes while we’re talking… maybe just start by just telling me about yourself.
J (JL): I was born in_____… left for ______ in 1981 till 1994 returned to Ireland and went to (University) at night time. I worked full time and studied part-time. I did that for 6 years… got my degree ……. and worked in the profession and after that it was a bit of mystery for me.
P: What do you remember?
J: How do you mean?
P: What happened to you?
J: What happened me? I don’t know.
P: What do you remember of your early life?
J: Oh yeah, I remember being brought up I remember everything up to about 2 years ago…3 years ago (*Suddenly the alarm goes off somewhere in the room and we both cannot find the source for a while, and analyst and patient share laughter*).
P: So you don’t remember anything after 3 years ago?
J: No
P: So what happened?
J: I have no idea.
P: And would you like to tell me about your life before 3 years ago?
J: I used to be a (professional position)…. (i.e., *JL had already forgotten that he mentioned his work.*)
J: I can’t read… I can’t get the information to stay in my head. And I don’t know why I could read and read and read and read and I would have to go back and start again… just have to keep repeating it over and over and it just won’t stay in my head (*He seemed to be aware of his memory problems, but it did puzzle him why this was so*).
P: And what about listening to audio tapes, watching TV?
J: I can watch TV, but ask me about half an hour of watching and I haven’t got a clue about what I’ve been watching so (*JL did not appear to be unduly upset about this*).
P: You can’t hold the information?
J: Yeah
P: It gets dropped.
J: Mmm mmm it goes in one ear and out the other
P: What is that like for you?
J: It doesn’t bother me at all… if there is a film on I will watch it and I will really enjoy it but don’t ask me about the film a half an hour after I was watching it. I wouldn’t even know if I was watching it or not.

JL’s clinical presentation during the following sessions offers additional information regarding the extent of his amnesia. When collected from the reception area, JL appeared not to remember the analyst’s name, the location of the consulting room where they have met the week before, nor the layout of the building. During the initial sessions he did not formulate questions regarding why he was meeting with the analyst, nor who the analyst was. Neither were there ever any explicit references to the previous session, suggesting some level of temporal discontinuity. Nevertheless, JL could bring back topics that had been discussed during the previous meeting, most notably sports, which seemed to have been ‘preserved’ somehow in memory. Interestingly, even though this topic was similar, the emphasis put by JL was different on each occasion. The repetition of certain topics across sessions was a cardinal feature of the therapeutic process:

J: Did you watch any sport over the weekend? (*This was a topic discussed during the previous session.*)
P: I didn’t…Did you?
J: No, not really. I did watch Rory McIlroy though.
P: Do you like golf?
J: Yes, I used to play it…but I was never really any good at it. That’s going back a couple of years now.
P: Did you enjoy it?
J: Oh yeah
P: How are things in Bellevue (his residential unit, not the actual name. Name is intentionally changed to protect anonymity)?
J: Same old same old… ticking along… (2 min silence) … Did you watch any of the golf just on there now?
P: No, I didn’t see any of it.
J: Rory McIlroy did very well. I don’t know what happened to Tiger Woods. He’s gone off the radar completely.

### Neuropsychological Presentation

JL’s premorbid level of general intellectual functioning was estimated to have fallen within the *average range* (*WTAR*, Wechsler, 2001). In contrast, his post-injury general intellectual functioning appeared within the *low average* range (Total IQ = 89, WAIS-III, Wechsler, 1998). There was no observable discrepancy between verbal and visual abilities (Verbal IQ = 88, Performance IQ = 87) and a normal performance on Verbal Comprehension, Perceptual Organization, and Working Memory Indexes from the WAIS-III. Only Processing Speed appeared below the normal range according to age and educational level (scaled score = 6).

Memory assessment confirmed the clinical presentation of profound anterograde amnesia, characterized by a marked impairment in encoding and recalling verbal and visual information after a delayed period of time. In the Logic Memory Task (WMS-III, Wechsler, 1997) immediate recall was moderately impaired (scaled score = 6) while delayed recall was severely impaired (scaled score = 1). A similar picture was observed in the deficit recalling information, both immediately (5/50 units) and after a delayed period of minutes (3/50 units). In the Rey–Osterrieth Complex Figure (Osterrieth and Rey, 1944), JL only managed to recall five elements in the delayed condition, thus suggesting a similar profile to the one observed in the Logic Memory Task. It is important to note that JL did not improve significantly his performance when cues were provided in the Logic Memory Recognition task (18/50), thus suggesting that his memory impairment was a consequence of a deficit in encoding (taking in) and consolidating (learning) new information and not retrieving -as is commonly observed in patients with dysexecutive syndromes after frontal lobe damage. JL’s adequate performance on executive tasks that assess working memory (*Spatial Span WAIS* scaled score = 12; Digit Span WAIS scaled score = 8) as well as planning, reasoning, and problem solving (Twenty Question Task scaled score = 10, D-KEFS, Delis et al., 2001) supports this conclusion. This ‘hippocampal’ profile of memory impairment was consistent with JL’s preserved ability to focus his attention and hold onto information for seconds, on its *immediate* presentation. However, JL’s difficulty encoding new information meant that any distraction during the task resulted in a failure to retrieve information after the interference.

## Psychotherapeutic Process

The psychotherapeutic intervention consisted of seventy-two, once-weekly, face-to-face, 50-min sessions of psychoanalytic psychotherapy. The therapy took place in a consulting room in a busy brain injury rehabilitation day service center. The room was used for neuropsychological and psychological consulting and was allocated to JL’s therapy for the same 1 h every week. The psychotherapeutic work was conducted by the lead author of this paper (PM), who is an experienced Psychoanalytic Psychotherapist.

The treatment was defined as a psychoanalytic psychotherapy since the intervention was informed by psychoanalytic theory and technique but did not take the same intensive form as psychoanalysis. We followed here the work of authors like Bateman and Holmes (1995); Etchegoyen (2005), who propose that any intervention that undertakes to first understand a situation by applying psychoanalytic principles, and to then formulate an intervention based on such understanding in a clinical context, is a form of psychoanalytic practice.

### Case Formulation

JL’s case is complex, since there are several factors that interact in his clinical presentation, particularly in the process of emotional adjustment to his brain injury. As described above, he presents with a profile of severe episodic amnesia, although other important cognitive abilities – such as executive functions – appear to be relatively spared. As can be observed from the vignettes presented, JL exhibits a clear awareness of his inability to perform certain mental operations (‘*I can’t get information to stay in my head’*), but he is apparently unable to link such problems with his brain injury, or to his amnesic syndrome (‘*I don’t know why’*). In addition, his emotional response to this situation is peculiar in that he is apparently indifferent to the existence of such deficits (*‘It doesn’t bother me at all’*). This neutral evaluation of his emotional deficits, technically labeled anosodiaphoria, has been frequently described in patients with episodic amnesia (for a review, see Tate, 2002).

This lack of concern about his deficits, and the impact they have on everyday life, is related to the paucity of JL’s *explicit* complaints. In fact, during the first sessions, the only preoccupation expressed by JL to the analyst is that he was not able to live independently. However, this only appeared sporadically, and JL did not seem proactive in continuing the discussion of this topic with the analyst throughout the sessions, nor finding help in understanding and solving this problem. On a more implicit level, this complaint appeared to be linked to his interpersonal difficulties with the staff of the residential unit. Thus, JL reported that he felt that all of his movements were monitored by the nurses – or the ‘sisters,’ as in ‘nursing sisters.’ Interestingly, his favorite films were *Escape From Alcatraz, The Great Escape*, and *Return to Alcatraz.*

P: It’s very difficult for you not having the freedom.
J: Not being able to do what I want to do when I want to do it, it can really get on my nerves sometimes, uh huh even just to walk to the shop or do something like that I can’t even just to go for a walk.
P: You can’t go for a walk on your own?
J: No … that really pisses me off.
P: There is a huge sense of loss around your freedom?
J. Oh there is yeah I don’t have any freedom now. Even when I was in (inpatient unit), I could go for a walk around the hospital… at least get some exercise.
P: And that can’t happen in here?
J: No they won’t even let me outside the front door without wondering where I’m going, who I’m going with and how will I get back …That’s the real big thing that gets to me at the moment.

Another source of conflict with the staff was the administration of his daily insulin dose. The ideal situation would be for JL to have his morning insulin roughly 10 min prior to breakfast. This would stabilize his blood sugars for the day. However, the timing of the insulin varied according to which members of staff were working. In consequence, more often than not, breakfast arrived first, and then JL refused to eat it, as this meant his sugars fluctuated throughout the day. This situation regularly brought JL into conflict with the ‘sisters,’ and this conflict, on occasion, could become quite heated and animated.

From a psychoanalytic perspective, the interpersonal conflicts presented by JL are clearly grounded in a present reality but may also be related to his early history of neglect, and to the personality traits that were defensively crystallized as a way of coping with such a neglectful environment. This is perhaps observed in the split relationship that JL establishes with the ‘sisters.’ In some situations, for example when the insulin is not provided on the right time, the nurses are experienced as neglectful and useless, and JL reacts to this with anger and frustration. In other moments, when the sisters are monitoring his behavior in order to keep him safe, he experiences them as controlling, and positions himself as someone self-sufficient that does not need any help. Apparently, there is here an interaction between the lack of awareness regarding his deficits with the exacerbation of a narcissistic pattern of defense. On this argument, his brain injury has disrupted his capacity to be autonomous, and he is now forced into depending on others, a position that has been rather traumatic in the past. In consequence, the ability to ask for help and accept help, something necessary in adjusting and coming to terms with brain injury, is seriously compromised.

### Psychotherapeutic Process and Transference

There is a small literature describing transference phenomena after brain damage (Goldstein, 1954; Pepping, 1993; Lewis, 1999; Kaplan-Solms and Solms, 2000; Coetzer, 2006; Salas, 2008; Yeates, 2009; Klonoff, 2010; Salas et al., 2013; Tiberg, 2014). However, case reports describing how this process unfolds when working with amnesic patients are few in number. In the only paper addressing this topic, Turnbull et al. (2006) suggested that profound amnesia did not compromise the *generation* of feelings toward the analyst. The case of JL is remarkable in that it offers, for the first time, supporting evidence not only to the fact that episodic amnesia spares individuals’ capacity to *generate* transferential feelings, but also their capacity to *modify* them through the use of analytic tools, in an apparently long term set of changes.

The transferential process between JL and his analyst might be considered as the evolution of a narcissistic transference. It can be roughly mapped into three phases (characterized below through specific moments) each of them exhibiting different degrees of permeability between JL and the analyst. These moments reflect different ‘scenifications’ in which JL and the analyst adopted distinctive positions, particularly in relation to core conflicts associated with depending on others and accepting the new vulnerable aspects of the Self after his brain injury.

#### Rejecting: The Liffey Bridge Incident

The first moment where strong transferential feelings emerged was early during treatment (session 6). JL’s usual routine was to arrive very early for therapy, often before the analyst. Notwithstanding his considerable memory impairments, sometimes JL took a walk alone from the consulting room premises, down to the quays of the river Liffey, and back up again. It was a relatively short walk, on the same street, with no turns or major junctions, a walk which took roughly 10 min. One day, JL asked the analyst what he thought of “the little pedestrian bridge over the river Liffey.” This question evoked confusion in the analyst, who knew the nearest pedestrian bridge was at least four or five blocks down the river, and thought that it was unlikely that JL could have walked that far and made his way back in 10 min. The analyst cautiously challenged JL’s assertion that this was a pedestrian bridge, and commented that he (the analyst) had in fact *driven* over the bridge on his way to the session. The session then took a difficult turn, since JL started holding firmly to his belief that this was a pedestrian bridge. The analyst later commented in his notes about this moment: “*There was a clear sense that he was clinging on to this understanding and any attempt to question it elicited a strongly defensive response.”* JL reacted as if the analyst was himself delusional, and said: “You couldn’t have, there is no way you could fit a car on that bridge, it’s just not big enough.” JL’s response evoked a sense of doubt in the analyst, who wondered whether there was another pedestrian bridge nearby which he had simply forgotten about. After the session, the analyst commented on this episode: “*I momentarily started wondering whether this was another manifestation of the amnesic countertransference, where I simply start to forget things just like JL.”* He also resolved to investigate this further on the way home. However, as was often the case with thoughts around JL, this resolution slipped from the analyst’s mind soon afterward.

The interaction between JL and the analyst can be summarized in the following main elements. Firstly, there is a disagreement between JL and the analyst. Secondly, the analyst’s questioning triggers a moment of emotion dysregulation in JL, which is followed by a defensive scenification: the analyst is stupidly wrong (‘you cannot fit a car on that bridge’), foolish and deluded. JL, on the contrary, is right and clever, and there is nothing possibly at fault with his mind. Thirdly, such scenification generates particular feelings in the analyst; he feels confusion, uncertainty, and starts wondering whether his mind is working appropriately or not. The analyst now feels foolish and deluded.

Theoretically speaking, it is possible to understand this episode as the defensive scenification, of a highly fixed narcissistic structure, which facilitates the regulation of negative feelings that emerge when acquiring some traumatic awareness about presence of severe cognitive problems. On this account, JL is not permeable to the influence of the analyst (i.e., the analyst’s alternative view of reality), and information cannot flow between them, and perceptions cannot be modified. In consequence, interpretations are experienced as frightening and threatening, with JL investing large amounts of energy in preserving his model of the world.

Defensive reactions like this have been described before in patients with brain injury, and are often referred to as narcissistic rage. According to Klonoff and Lage (1991) the rage is an external representation of the internal shame experienced as a result of narcissistic injury. To Kohut (1972), rage and shame are the two principal experiential and behavioral manifestation of a disturbed narcissistic equilibrium. Narcissistic rage would refer to aggressive responses that aim to destroy the offending self-object, when this self-object is experienced as threatening the continued cohesion or existence of the Self (Wolf, 1988). Shame, on the other side, arises when the ego is unable to provide a proper discharge for the exhibicionistic demands of the narcissistic self or when the self-objects do not respond with expected mirroring (Kohut, 1971). In this case, the analyst’s offense is to point to JL’s mistake, an action that JL perceives as an attack to the fragile integrity of his mind. An interesting element of this case, which is novel in understanding the phenomenon of narcissistic rage, is the projection of parts of the Self *to* the analyst. In this case, it is the *analyst* who experiences confusion, uncertainty, and also doubts about the proper working of his own mind. And it is also the analyst who experiences a decrease in self-esteem, by feeling foolish and stupid (shame!). One conclusion might be that by considering the impact that narcissistic rage has into the analyst, a more relational paradigm can be constructed, where elements of the Self that cannot be thought by the patient can then be contained and metabolized by the analyst.

The incident of the bridges over the river Liffey was a cornerstone of the therapeutic process, in that it allowed the analyst direct access to JL’s experience of fragmentation, when in touch with his amnesic mind. It also contributed to understand the nature of the defensive mechanisms that he deployed when attempting to regulate such states. The narcissistic nature of his defenses also illuminated interpersonal conflicts that occurred outside therapy, which might be linked to his history of family abandonment. For example, JL’s interpersonal difficulties at the center were marked by the belief that he did not need care from the sisters, for he did not need *any* help after his brain injury. He felt that he could manage by himself. However, when depending on others was out of his reach (for example the daily insulin injection), their failure in providing *exactly* what he expected triggered intense reactions of frustration and rage.

This incident is also relevant for a more theoretical reason. After the episode, JL spontaneously brought the “bridge” incident into four consecutive sessions, something rarely seen during the whole treatment. He often began the sessions by asking the analyst “Well, did you get a chance to check out that bridge?” This phenomenon is particularly interesting in that it offers evidence to support the claim that patients with deep episodic amnesia can encode and retrieve new memories. Furthermore, it also suggests that the emotional nature of the event might be key in the registration of this new information, and that intense emotional events appear to enhance the often compromised temporal continuity in deeply amnesic patients, presumably through rehearsal. Notably, in a life of daily failure, the bridge incident is (from JL’s perspective) a personal triumph – a moment when he is more knowledgeable than the analyst. The analyst wrote in his notes about this particular event providing a temporal formulation: “there is a strong desire for JL to make sense of this puzzling experience, and to use the therapy, and my mind, to make sense of what happened.” Considering the previously described defensive scenification, this repetition appeared to open the possibility of a new form of relationship. By using the mind of the analyst to make sense, JL was beginning to accept placing himself in a position of necessity, and also to tolerate the fact that he might not be able to solve the puzzle on his own. A new interpersonal configuration, based on care and collaboration, then began to emerge, modifying the previously installed ‘clever-stupid’ script.

#### Starting to Take in: “I Know What You Are Saying, But…”

A second phase of the transference was characterized by a progressive loosening of JL’s narcissistic structure, with a higher degree of permeability to the analyst’s interventions. JL was able to use the analyst more consistently, in order to explore the impact that his mental dynamics had on the way he approached problems in the outside world. However, even though information was exchanged more easily between JL and his analyst, and JL was more prepared to consider the analyst’s point of view, there was still resistance to take his suggestions in, or use them in order to modify his internal working models. The following extract, which belongs to a session from the middle part of the therapy (session number 39), illustrates these dynamics and possibly also symbolizes JL’s post-brain injury mind: an apparatus that seems to work perfectly (the TV) but is unable to access areas of the mind that store and process pre-recorded experience (the DVD player).

J: Yeah… (silence 6 min)… they got us new TVs there last week, last Wednesday. I got my new TV, the thing is not worth a bollocks. I just want my old one back now… my old TV, I could hook my DVD player in and watch DVDs on it and everything, the new TV I can’t.
P: Why not?
J: I don’t know why… it won’t recognize the TV is there.
P: You’ve got the right leads and everything for it?
J: Oh yeah it’s actually the same connections go from this TV into the old TV. Everything is exactly the same.
P: That’s very strange.
J: It just comes up as though it isn’t registering it.
P: And is there anybody there that can have a look at it for you?
J: I’ll have to get maintenance down… but I can’t see what they can do that I can’t do. You see what I mean?
P: Mmm
J: ‘Cos everything is exactly the same, same connections, same wires, same ports, everything … click it all on, all good, click favorites and nothing comes on.
P: You’ve tried turning it on and turning it off, all that kind of stuff?
J: Yeah
P: It’s very frustrating.
J: Mmm mmm …. Bring me back me old television and I’ll be happy.
P: Can you do that? Is it around?
J: I don’t know.
P: There’s nothing wrong with it by the sounds of it.
J: It was perfect.
P: What’s different about this new one? Why was it replaced?
J: I don’t know, no idea.
P: So something that worked perfectly well has been replaced or changed.
J: With a broken one.
P: With something that is broken.
J: Yeah
P: And you have no choice in the matter.
J: No
P: There is something that is not compatible, something is not working and you don’t know what it is.
J: I don’t know what is wrong with this one.
P: Internally?
J: Yeah. Because the remote control is exactly the same. Everything is exactly the same. It just won’t register that the DVD player is there…. I don’t think it is the DVD player, because the DVD player still works on the big screen on the TV inside.
P: It might be a flaw in the TV?
J: It must be it must be something wrong with the TV. How would you break a TV, but not break it so it looked like you broke it?
P: Laughs
J: Laughs… And get the old one back…that’s what I was thinking of last night. How am I going to wreck this TV, the new TV?
P: But is it not broken already? If it’s not picking up your DVD player, it’s not working.
J: Yes. There’s probably something wrong with it I can get all the channels, I can watch TV on it. But I don’t want to go to N and say I want a new TV, because this one is not working properly. She’ll be like “what’s wrong with it?”
P: Have you told her about the TV?
J: No
P: Why not? What stops you?
J: What am I going to get out of her?
P: She might help, she might be able to fix it?
J: No, I couldn’t see that happening.
P: Or have it arranged to have it looked at by somebody who might fix it… you rule out that possibility?
J: Yeah. Because it is not broken as such, you know, that sort of way.
P: It’s hard for you to ask for help?
J: Who, N?
P: Mmmm
J: Ah, yeah
P: You might explain to her that you’ve tried and ask her would it be possible to get someone to look at it. She might be able to arrange that?
J: I suppose that is one way of going about it.
P: It is hard for you to even entertain that possibility? It’s kicked to touch straight away.
J: Well in a way it is, because going to N and telling her that the DVD player is not working on my TV. She would be like so what! What do you want me to do about it?
P: And how do you know that?
J: Because that is N. That is what she is like… I’ve been through that with her I know exactly what she is going to say.
P: Can you think of a way of saying it… ?
J: I’ve been wrecking me head trying to bring it up with her, like I can’t watch my DVDs now because the DVD is not working or it isn’t compatible or what ever.
P: I’m just thinking about the way you are thinking about it. You are being confrontational with her.
J: Yeah
P: You want to confront her about it, about why the TV is not working?
J: Mmm mmm.
P: Where a better approach might be to ask her for help… You know, “Would you mind having a look at it for me?”
J: What is she going to say? I don’t know anything about it!
P: I think if you bring it to her, in a way, where you accuse her…
J: In a way that she is involved.
P: Or giving out about wanting your old TV back?
J: I know what you are saying.
P: Immediately it is going to have a defensive reaction … Maybe it is about having a conversation about it… asking for help … It might be a way of getting her involved in a constructive way?
J Yeah… Either that or just give me back me old TV please!
P: I think it taps into something we were speaking about before the Bank holiday, about how it is hard for you to investigate things for yourself, and ask questions yourself around your own situation in X, and the possibility of getting resources around accommodation other than X?
J: Yeah
P: It’s difficult for you to put that thinking in place around it for yourself, about how you might be able to help yourself?
J: Mmm mmm.
P: Like with the TV, the initial reaction is to get angry and to confront someone?
J: And lose the plot
P: Yeah… Where there is another way of thinking about it, which may help you, and serve your needs better.
J: Mmm mmm. Yeah, sure isn’t that just the long way around it all though?
P: In what sense?
J: Basically I know what she is going to tell me before I even ask her.

This extract from a session is interesting for several reasons. Firstly, it clearly shows a move from rejecting to taking in. In the first phase of transference, there was no space for difference between JL and analyst, and reality could only be interpreted from JL’s point of view. Here a more dialogic pattern can be observed, where JL and analyst can disagree, without endangering the therapeutic alliance. Secondly, the core conflict of the patient, his difficulty to place himself in a position of necessity, occupies the center of the analyst’s therapeutic efforts. In this case, however, the conflict points at events that are taking place out of the session, where other significant events or objects (e.g., N, one of the ‘sisters’) are the object of transference. In this particular situation, something important to JL is not working – something that gives structure to a big part of his everyday routine. A new TV has been placed in his room and it does not connect properly with his DVD. He has tried to make it work himself, without success. When facing this problem, he adopts a narcissistic position (I can’t see what they can do that I can’t do) which leaves him in a dead alley.

The whole session revolved around the efforts of the analyst to help JL gain some insight into this dynamic; to help him understand the mechanisms by which he gets angry and confrontational toward N, before even asking for help. It appears that early patterns of relating may have been transferred to N, who is fixed on a role that has no room for novelty (*Basically I know what she is going to tell me before I even ask her*). On a more implicit level, it is possible to suggest that JL’s behavior may be related to the certainty that his needs will not be heard, that they are not important, and that there is no other possible outcome. Even though JL can rationally *understand*, and somehow accept the logic on the analyst’s interventions, he does not *feel* the story can have a different ending. It is also interesting to note here that the level of negative emotion experienced by JL may also have a role in the rigid way in which he perceives reality. In the last part of the session, he comments that when he gets angry he ‘loses the plot,’ suggesting by this that his capacity to symbolize is compromised by negative feelings. A question that emerges from this observation is whether there is an interaction between negative arousal and cognitive impairment. In other words, is it possible that in moments of negative arousal, patients with cognitive deficits become more prone to default to primitive forms of transference?

#### Full Use of the Analytic Space: “I’ve Got to Stop Losing Things”

A third phase in the evolution of transference could be observed toward the end of treatment. At this stage, JL appeared to have moved away from a rigid narcissistic position. As it can be observed in the extract below, JL was now more permeable to the analyst’s interventions, and the therapeutic dyad could be described as one with a reciprocal and unhindered flow of ideas and experiences. More importantly, JL appeared more open to the possibility of altering his internal working models, which translates into interpretations being more completely taken in and used, to make sense of his personal story and brain injury.

J: Now I’ve got to stop losing my glucometer.
P: You’ve lost it again?
J: (Silence 1 min)… I was watching that program about the Caribbean cops. Did you ever watch that?
P: No?
J: It’s about cops in the Caribbean and they have to go in and raid places for drugs and all that… God you want to see this…
P: It’s a documentary?
J: Yeah. Its real life, about cops in the Caribbean, and they broke into this warehouse, and the amount of grass that they got was unreal.
P: In the Caribbean?
J: Yeah. Stag parties and all that. The amount of drink… that is drunk. And they hire a boat and the boat takes them out to sea, and everything on the boat is free once you pay for the boat – all the alcohol is free on the boat. And the English were drinking and drinking and falling all over the place, falling overboard and everything. Madness it was. What was the driver saying, three-hundred quid for the boat for the day. Three-hundred quid between 10 of you is nothing, and as much as you can drink a tenner a piece to go out and get pissed and do whatever it is you want to do out there.
P: And these two pieces were on the TV last night?
J: Mmm mmm
P: About the alcohol and the marijuana?
J: Mmm mmm. Yes… Caribbean cops it’s called … You want to see the sniffer dogs at the airport, watching people going back. The amount of grass that is confiscated, and people getting fined and all that going to jail and everything ludicrous. Some guy was caught with two big suitcases full of grass trying to get it back… Crazy, you know what I mean? No clothes. No nothing else in the suitcases just grass how he expected to get them through I don’t know… …. It was ridiculous. Two thousand pounds he had paid, and he reckons he would have made three hundred thousand pounds from it… Now he’s serving time in a Caribbean jail.
P: … I’m just thinking about what comes into the session today. The excess alcohol… The excessive use of alcohol, the huge amounts of marijuana and the consequences of engaging with that… The cops are involved, there is punishment, and somebody ends up in jail… I am just thinking about that in terms of your own experience of alcohol and marijuana use and your diabetes and the subsequent brain injury?
J: Yep
P: And how that might feel the same. That you are being punished for that.
J: Being stuck in jail.
P: Yeah. It might feel like that for you… And you talk about Oscar Pistorius (earlier discussion in session). Does he get off? Does he get a reprieve? It’s very like the situation you are in at the moment. Are you going to get out of Bellevue? Will you get off?
J: Mmmm mmmm
P: It’s very much on your mind, these things about the past, and where it has led to, and where you are now and how will you get out of it.
J: That’s true.
P: In a couple of different senses. The physical sense of being in Bellevue, but also the psychological sense of your mind and the damage to your memory.
J: How do you mean?
P: Will it recover? Will you recover? Will it improve? Will it change?
J: Well I’ve been told there is a great improvement, but I can’t see it because I am living with it day in and day out.
P: So someone told you there was an improvement?
J: Oh yeah
P: This was the neurological tests you had recently?
J: Oh yeah… I can’t see any improvement I didn’t see anything wrong with me in the first place. So I didn’t know any better.
P: Ok… so what to do you think now? What’s your sense of how things are now?
J: How do you mean?
P: Memory wise
J: I think now my memory is a lot better that it used to be. Mmm mmmm. Now I can remember people’s names, and put people’s names to a face, and all that sort of craic.^2^ Because beforehand I couldn’t’ do that.
P: So it has improved, in that sense and somebody tells you, the psychologist who ran the tests, tells you there is an improvement, and it is hard for you to take it in.
J: I couldn’t see it, no.
P: And at the same time you are aware of your memory being better than it used to be?
J: Oh yeah. When I look back on things, I can remember now, and I see I am a lot better than I used to be.
P: And what is that like?
J: I don’t know… To think back about how I used to be… I don’t know… I don’t want to go back there… put it that way.
P: Ok.
J: (Laughs) Now if I could only remember where I’d put my glucometer I would be a happy person. For the life of me I couldn’t find it can’t find it anywhere.

This vignette is a good example of the ease of movement between analyst and JL during the last part of the therapy process. In general, JL appears less defended, which translates into a more proactive and productive chain of associations. JL is not afraid to bring things up, and is less likely to reject the possibility of exploring the different perspectives offered by the analyst. Another way of thinking about this is that JL’s capacity to tolerate what the analyst has to say has developed, to a point where he can hold new perspectives in his mind and use it to symbolize new possible meanings.

Another interesting element, during this last phase is the more open *elaboration* of conflicts, particularly the ones around his loss of independence. This can be observed in the analyst’s effort to link JL’s associations (drug abuse, sentence, jail) to his personal history. It is interesting that JL does not immediately reject this avenue of exploration, but accepts it as a *possible* interpretation. This shift is particularly important in that it allowed the progressive elaboration of feelings that were, initially, difficult to tolerate (anger and guilt for not taking care of this health) and needed to be directed to others (e.g., anger toward the sisters). A possible consequence of such shift may well be JL’s disposition to talk more openly about his memory difficulties, which can be considered an acknowledgment of their existence. This picture differs quite importantly from the initial phase of treatment, where very rigid and primitive defense mechanisms were needed to avoid contact with this painful reality.

### Countertransference: One or Many?

Countertransference is a long-standing theoretical and technical concept in psychoanalysis (for a review, see Thomä and Kächele, 2010). However, its consideration when working with brain injured patients is quite rare (Pepping, 1993; Lewis, 1999; Kaplan-Solms and Solms, 2000; Salas, 2008; Yeates, 2009; Klonoff, 2010; Salas et al., 2013; Tiberg, 2014). Lewis (1999), using ideas from Racker (1960, 1968), has offered perhaps the most comprehensive review on the topic, suggesting four possible configurations: (a) *complementary* (to feel what the patient’s significant others have felt in the past in response to the patient’s behavior); (b) *concordant* (to feel what the patient is feeling, or what the patient is struggling not to feel); (c) *idealizing* (to feel admired by the patient or seen as an omnipotent figure); and (d) *mirroring* (to feel the need to accept and confirm the patient’s Self, to become an extension of the patient’s Self).

Most of the countertransferential feelings experienced by JL’s analyst can be readily described as examples of the configurations proposed by Lewis (1999). For example, during the episode of the Liffey bridges, the analyst’s countertransference can be considered as *complementary*, as he became captured by feelings (e.g., confusion and worthlessness) that JL apparently could not tolerate. Similarly, the analyst reported occasional strange bodily sensations, which took the form of a dizzying semi-nauseous feeling, tinged with confusion. This strangely visceral and somatic feeling was at times subtle, and at other times pronounced. However, it was virtually always present at a background level during the sessions. The nauseous or dizzy feeling was eventually understood by the analyst as a possible somatic correlate of a ‘hypo-glycemic’ event, which JL feared and talked about regularly during sessions. On other occasions, the analyst experienced countertransferential feelings that were *complementary* to the patient’s own mental states. For example, during the Liffey bridges incident the analyst felt stupid and incompetent, a common experience for all those caring for JL (e.g., sisters). This type of countertransferential feeling appeared to be linked also to the analyst’s own insecurities about his ability to deliver psychotherapy to such a complex patient.

Lewis (1999) also comments that, besides the classic countertransference configurations observed when working with brain injured patients, the analyst may experience feelings in response to his/her perception of the patient’s deficits, and the patient’s reaction to his own deficits. The case of JL is interesting in this respect, in that it suggests that the patient’s deficits themselves (not its perception or reaction to them) can also influence the analyst’s mental states. This phenomenon has been referred before as ‘*organic’* countertransference (Salas et al., 2013), and appears to be produced by the neuropsychological attunement between analyst and patient. From a Kohutian point of view, this can be considered as a form of *merging* countertransference, where the analyst becomes an extension of the patient’s self (Kohut, 1971). JL’s analyst experienced several episodes where this type of countertransference appears to be in action. Changes in the analyst’s memory functioning is perhaps the most common example, with forgetting names, words or places as a recurrent phenomenon. The analyst describes (in his notes) that during the sessions it was rather common to find himself and JL unsuccessfully searching for the name of a soccer player, sporting event, movie or actor. From the analyst’s perspective, these memory gaps were impossible to address during the session itself, but became far more accessible the further away in time and space the analyst became from the session. It is also interesting to note that these memory ‘slips’ did not only occur during the analytic sessions, but also around them, in logistical and practical dealings with the institution that took care of JL, or with the residential unit at which JL lived. Events, appointments and notifications between institutions were often dropped, missed or confused. This appears to be the first time that such memory findings have been formally reported in working with amnesia, but we suspect that the may well be common (but underreported).

## End of Treatment

As it was noted in the Section “Introduction,” one of the main limitations of previous studies exploring how patients with profound amnesia present during psychoanalytic treatment has been their short duration. In the case of the study by Turnbull et al. (2006), for example, the patient was seen only eight sessions, thus only allowing observing the *emergence* of transferential feelings, but not considering the *course* of such feelings during treatment or their *elaboration* at the end of treatment. The case of JL is unique in this sense, by offering for the first time evidence regarding the impact of profound amnesia in the dynamics of the termination stage.

The most important observation regarding the ending of psychotherapy is the presence of a similar amnesic pattern to that witnessed at the beginning, and also during the course of treatment. This pattern was characterized by the absence of ‘explicit’ recall of the fact that treatment was ending, apparently together with a level of ‘implicit’ and emotional awareness of the situation (see below for examples). It is interesting to note also that this pattern influenced the analyst’s countertransference during this stage, particularly in relation to his own experience of separation and mourning. The description of these phenomena, and its theoretical and technical implications will be addressed in this section.

### Are We Ending? A Dissociation between Explicit and Implicit Awareness of Termination

The amnesic pattern presented by JL along the course of treatment involved an absence of explicit recall that the treatment was ending together with, an implicit awareness of the impending separation and loss. The lack of explicit recollection can be appreciated in two ways. Firstly, during the last period of treatment, JL was always surprised when the analyst brought his attention to the fact that therapy was ending. Secondly, JL never enquired the analyst, at least spontaneously, regarding termination. The following vignette from the penultimate session offers an example of how this phenomenon was observed during the termination phase. In addition, it also illustrates how JL’s difficulty to stay on topic, without ‘dropping it,’ impacted the elaboration of feelings related to termination:

P: And have you thought about the sessions ending in here?
J: When are they ending?
P: Next week is our last session.
J: Is it?
P: Yes. (silence 1 min)
P: What are your thoughts about finishing up?
J: I don’t know…what am I going to do on a Monday afternoon now? How long have I been coming here? Just over a year? A year and a half?
P: It has been 2 years.
J: Two? (silence 2 min) (there is a squeaky sound coming from outside)… we need some WD40. What is it?
P: I have no idea.
J: So, have you been to the cinema lately?
P: No I haven’t.

Most of the sessions during the termination phase began in the same way, with JL arriving to them oblivious to the fact that the treatment was ending. The analyst writes about this phenomenon in his notes: *Each session involved me repeating the same question about the ending arriving and enquiring about J’s thoughts on this. Each time the reply was the same; as if it were the first time to hear about this.’* Even though JL appeared unable to consolidate information about the impending termination, it is extremely interesting that his associations during the last sessions were often related to issues of death or loss of objects. Consider, for example, the following extract from one of the last sessions, where JL comments on his lost glucometer pack, where he kept important cards and documents. The loss of these, and how to replace them, figured frequently in the sessions leading up to the final of treatment. JL was pre-occupied with its whereabouts and retracing his recent movements with the glucometer was a common line of associations:

J: You know your Personal Public Service Card?^3^…Who do you get them off?
P: Revenue Office, I think?
J: Do you have to fill out a form or something?
P: I would say so.
J: Because I’ve lost mine.
P: It is with your glucometer, is it?
J: Yes
P: Have you said it to anybody? To your key worker? To M or C?
J: You see I don’t know where I lost it … You see it wouldn’t be here, if it was it would be in my lunch bag, and my lunch back is back in Bellevue hospital.
P: Have you asked at reception?
J: Here? Oh no. It’s not here, I know that.
P: Oh, you know that… You have mentioned that you were in B___, and that you were playing snooker recently.
J: No, I don’t take my machine with me.
P: Oh you don’t take it with you? (*silence* 1 min).

JL’s associations during the last stage were not exclusively related to the loss of concrete objects, but also included thoughts and memories associated with death and loss. For example, JL occupied considerable time talking about a resident who had recently passed away, and whom JL had known since he was hospitalized after the brain injury. Memories from childhood, also related to loss and its elaboration, emerged during the last sessions. A particular one is interesting, since it evokes Freudian ideas (Freud, 1950) on separation (fort-da):

J: … I used to kick the ball out over the cliffs, and the ball would come straight back to you. I never lost a ball over the cliffs … Ahh yeah everybody used to walk down the cliffs from the cottage we were staying in… I always wanted to run and jump off, to get pushed back by the wind, but I was never allowed to. It always amazed me you could kick a ball as hard as you could out over the sea and it would just come floating back… They parasail off it now, they go parasailing off there.

The theme of succession also featured as part of JL’s associations during the last sessions, with an emphasis on what will happen to him after the ending, and who will occupy the analyst’s place. The analyst picked up on the potential meaning of these associations, and offered them to JL, as material to reflect about the ending of treatment.

J: What do you make of Nelson Mandela?
P: He’s not very well at the moment is he?
J: No, I’d say he’ll kick it before the Queen of England does.
P: He’s critical at the moment. He went back in to hospital yesterday.
J: Did he? I’d say it’s a bet between himself and the Queen who’s going to die first. I know who Charlie has his money on. He would be saying “come on hurry up” … I can’t imagine King Charles being anything or doing anything. King Harry, Now he’s the one I would like to see in power. I reckon he should knock his brother off.
P: He’s next in line is he?
J: He’s third in line. It’s Charles, then his brother, then him.
P: Ok
J: Yeah, he’s the youngest of the boys. Christ! Imagine the country if he got into power. Have you ever seen him on the TV programs?
P: King Harry or Prince Harry?
J: Prince Harry. He’s a wild kid. Always giving the secret service or whoever looks after him the skip. He goes to night clubs and everything. Yeah, sure, he went off to join the marines and went to Germany and all that. So what happens when Charles dies? Camilla wouldn’t’ become Queen?… his wife?
P: I don’t know.
J: It must be the eldest son or the eldest daughter in their family, so Charles must be the eldest of the queen’s children and Philip is his eldest… (*silence*, 1 min) Are you watching any of the athletics that’s on TV at the moment?…
P: I am just thinking, this idea about succession that is on your mind. About the King and who succeeds him and so on and so on… and I am just thinking about that in the context of what is happening here in the therapy today. It’s the last session and maybe that is on your mind what will…
J: Follow on from it.
P: Follow on what, or who will be the successor of the therapy?
J: Mmmm… That’s true.
P: I think that you may not have remembered that today was the last day of the therapy. I’m not sure had you remembered.
J: No
P: But I think that in some part of your mind it was registered and it came in here (s*ilence*, 1 min). I am just wondering what is it like for you?
J: I’m just thinking, what I am going to do on a Monday afternoon? Play bingo?
P: …. What are your thoughts about finishing?
J: I don’t know. I am going to miss you… I have got to get your address to post you on that picture (*a painting of the forest that J has been working on*), (laughs). Now don’t expect it yet for the next couple of years but I’ll post it on.

### Am I the Only One Mourning Here? The Impact of Amnesia on the Analyst’s Countertransference during Termination

It has long been noted –based on the observation of neurologically healthy individuals– that the elaboration of countertransferential feelings during the end of treatment is a particularly difficult challenge for analysts (Schlesinger, 2014). However, an interesting question that remains to be addressed is whether brain damage adds further complexity to such elaboration. Notably, whether cognitive impairment may impact the mind of the analyst, and generate quite unusual countertransferential feelings. As noted above, this type of response has been referred to as ‘organic countertransference’ (Salas et al., 2013), since its source is not the patient’s conflicts, but the particular way in which the mind of the patient functions after the injury. Analysts that work with individuals that have acquired a brain injury often struggle to disentangle whether their countertransferential feelings are a response to patients’ cognitive deficits or conflicts. The case of JL offers some tentative answers on this matter, suggesting ways in which, during termination, countertransference is fed by both the impact of neuropsychological deficits and the influence of patient’s dynamics. Here, we briefly address this problem, describing the analyst’s countertransferential experience during termination, and his difficulties in using such experiences as a source of information upon which to decide how to proceed analytically.

What is the impact of profound amnesia on the countertransferential feelings generated during the termination stage? How does the patient’s total lack of recollection regarding the ending of treatment impact on the analyst’s feelings and interventions? In the case of JL’s analyst, this was a constant dilemma during the last phase of treatment. The analyst comment about this in his notes: *‘Ought the analyst to deviate from the usual patient-led flow of the session, when the ending was imminent, but there was no indication of JL having remembered this? Or is there an obligation on the analyst to remember this for the patient and bring it into the session?* After another session the analyst comments: ‘*20 min into the session and with no explicit reference to the imminent ending of the therapy, the push and pull to act within me became too great to bear. This was a push and pull between the idea of whether or not to move out of position, and bring up the subject of the ending of the therapy, or to sit with the material that was coming and try to analyze it.*

To the analyst this dilemma was not simply a technical decision that needed to be made, regarding whether or not remind JL about the ending, but also implied becoming aware of strong feelings that gradually emerged along sessions: ‘*An increasing sense of obligation rose within me, a sense of obligation to JL, to do something for him that he was unable to do, perform the function of remembering for him. It felt strange, as I had performed that function for him in many ways over our 2 years of working together. This felt different. It was very difficult for me to remind him of our time together coming to an end. I had of course done this several times already in previous sessions leading up to the ending, however, I noted an increasing sense of disappointment and frustration growing in me the closer we came to the ending. I understand this as being the gradual realization that JL could not cognitively remember the fact that the therapy was coming to an end. Along with the disappointment and frustration I felt, I also experienced a terrible sense of powerlessness and despair.* After another session, the analyst keeps writing on this topic: ‘*eventually over time an ever increasing sense of sadness, annoyance and frustration grew in me. In the final sessions I noted what seemed like a hardening or intolerance, toward what J had to say, being present in me. I was becoming angry with J for not remembering. Later I came to understand this in several ways. One is the genuine sadness, annoyance and frustration in me about having to end the sessions due to lack of funding and the unsustainability of the therapy from the perspective of time and financial commitment. Another is that ending in this way is contrary to my usual practice, where I clearly state with patients that ending therapy is their prerogative, so the enforced nature of this ending was very uncomfortable for me. There is also my loss in the ending of these sessions for the relationship that had developed between J and me. I was going to miss him.*

It is interesting to reflect here on the emotions that the analyst is experiencing, as well as the possible sources of his grief. He comments that disappointment, despair and anger come in large part because of the realization that JL will not remember the end of treatment. However, he also links feelings of sadness and frustration (and perhaps guilt) to the unilateral way in which the treatment was terminating. A complementary interpretation is that his frustration and disappointment could be related to the realization that JL may not remember *him*. That once they separate, he will no longer have a place in JL’s mind. He will be – perhaps completely – forgotten, as happens with any new event or any new bit of information. This is perhaps also an important source of grief: JL will forget the analyst, as with everything else. Most analysts mourn their patients, helped by the conviction that, in some way, aspects of themselves will be preserved –or incorporated– by patients after treatment is ended. This is perhaps a similar feeling to the one parents experience with their children. It does not matter whether we die, something will pass on in them. Thus, the inability to consolidate new memories in amnesic patients may trigger in the analyst strong anxieties regarding separation, which may complicate the elaboration of the mourning process that separation entails.

## The Structure and Dynamics of the Therapeutic Process

An important issue that needs to be considered, when working psychoanalytically with brain injured individuals, is the impact that a specific profile of cognitive impairment may have on the structure and dynamics of the therapeutic process (e.g., Salas et al., 2013). If we consider the Generic Model of Psychotherapy (Orlinsky, 2009), the structure and dynamics of the psychotherapeutic process can be described as a set of interrelated dimensions: therapeutic contract, therapeutic operations, therapeutic bond, Self-relatedness and in-session impacts. It has been noted by Orlinsky that these five therapeutic dimensions acquire different configurations –temporal patterns– as the process changes over time – perhaps, as *micro-events* within therapy sessions, or as *macro-events* over the course of the treatment. The most important observation in the case of JL appears to be the disruption of these temporal patterns, compromising the continuity of micro-events (intra-session) and macro-events (inter-session). More simply put, profound amnesia impairs the individual’s ability to experience each analytic session as *one* temporal unit, and also compromises the patient’s capacity to connect elements that belong to distant moments in the therapeutic process.

### Intra-Session Temporal Discontinuity

Early on during the treatment the analyst noted an intriguing feature in his interaction with JL. Every session, after a couple of minutes, there was a sudden disruption, where JL stopped talking and appeared to withdraw from the conversation. This disruption was often followed by a pause that could last from a few seconds to a couple of minutes, where JL stayed silent. Then, JL appeared to re-initiate the conversation, without resuming the previously discussed topic, but returning to associations related to his past life, or to topics that were a shared interest with the analyst (typically sports). In the analyst’s notes, these events were described as a ‘memory reset’ and ‘dropping contents.’ Both images appear to portray the analyst’s experience that something had occurred in JL’s mind, something related to his memory impairment, which forced his mind –and also the interaction– to ‘reboot.’ Both metaphors –the ‘reset’ and the ‘dropping’– point to a process that is abruptly disrupted, and appears to re-start from zero. It is important to note that this ‘dropping’ of subjects occurred largely on an explicit level, since there were times where an underlying continuity in the apparently unconnected associations that emerged after the reset might be observed –or has at least suggested or intuited.

Regardless of this issue, memory reset posed important challenges to the analyst’s technique. Firstly, the intra-session temporal continuity was regularly disrupted, so that interventions based on linking contents from different moments inside the session appeared to be compromised. Secondly, interpersonal attunement was also interrupted, since the analyst’s following of the patient’s chain of associations came to an abrupt halt, causing counter-transferential feelings of confusion and perplexity at the beginning, and frustration later. An important central challenge for the analyst in the management of these events was to determine whether they represented a defensive maneuver that occurred when in contact with emotionally difficult contents, or whether they were the consequence of JL’s inability to consolidate new memories.

The process by which the analytic dyad re-engaged after the memory ‘resets’ is also of technical and theoretical interest. After the ‘pauses,’ JL often addressed the analyst with questions related to shared interests. It was not unusual, for example, for JL to ask the analyst three or four times throughout the session: “Did you watch any sport over the weekend?” or “Have you seen any good movies lately?” As can be expected, when these questions are formulated by JL, he has no recollection about asking the same question before, or whether they have been answered or not. The analytic notes reflect on this mechanism: “These shared themes are places of refuge during difficult periods in the sessions. After his memory has reset, these facts are used to anchor or ground JL Memories about mutual interests are presented like islands in the sea of uncertainty that constitutes the majority of the sessions. They form a safe ground after being shipwrecked by the failure of memory.”

Notably, these ‘restarting’ topics are far from random, and have clear interpersonal benefits for the relationship with the analyst. Thus the repetition of these questions might be considered as a regulatory mechanism. From an intrapersonal point of view, they offer mental contents that can be used to re-start the dyad-system, thus bringing some sense of direction and certainty. From an interpersonal point of view, these contents have also a regulatory role, since they facilitate the re-attunement of patient and analyst. They are *safe* areas, for JL knows that these topics are dear to the analyst as well, who is keen to re-connect with him, through them.

### Inter-Session Temporal Discontinuity: There is Novelty in Repetition

Profound amnesia not only compromises individual’s capacity to experience the analytic session as a unit, but also the generation of links between sessions, or what Orlinsky (2009) calls macro-events. The implications of this impairment for psychoanalytic technique are not minor, since meaning is often generated through the elaboration of elements from different sessions, and also through the elaboration of material related to the real life that occurs in between sessions. This capacity was markedly impaired in JL, who rarely connected the material discussed during one session with previous conversations, or with events that occurred outside therapy. This deficit is interesting in the light of a growing literature suggesting that individuals with profound amnesia experience important difficulties in moving backward and forward in time (e.g., Klein et al., 2002; Hassabis et al., 2007), a psychological process commonly known as mental ‘time travel’ (Suddendorf and Corballis, 1997; Suddendorf, 2013). It is possible that such time travel is a key psychological ability for the capacity to link elements from disparate moments in time, important when elaborating meaning during a psychotherapeutic process.

It is interesting to note that JL’s difficulty in linking material between sessions was not complete. During the treatment there were a small number of occasions where he did spontaneously bring material into sessions that had been previously discussed. A distinctive feature of these very rare episodes was that the material remembered by JL, and actively brought to session, had strong emotional content. The best example here is the episode of the bridges over the river Liffey, where JL brought the topic back to session four times. This observation is relevant for two reasons. Firstly, it supports the idea that neuropsychological deficits are not static, for they can be modulated by contextual elements (Bowen et al., 2010), in this case by the level of emotional salience. Secondly, because it suggests that mental time travel, or the capacity to move backward and forward in time, is a cognitive process mediated by important *non*-cognitive factors (D’Argembeau and Van der Linden, 2006).

JL’s temporal discontinuity between sessions posed important challenges to the analyst’s technique, forcing some adjustments. Perhaps the most important was a change in the analyst’s focus of attention: from the explicit contents brought by JL, which were often scarce and repetitive, to the implicit connections that underlie such contents. This shift allowed the analyst to spot the recurrence of emerging themes that appeared related to JL’s conflicts. Of particular interest here was the reconsideration of the therapeutic value of contents that JL repeatedly brought to session. For example, two very common repetitive topics brought spontaneously by JL were sports and cinema – described above as a way of re-booting the conversation. At first, the analyst considered this repetitive event as some form of mental perseveration. However, when carefully considered, repetitions were never the same, or they never ended in the same thread of associations. In other words, there was always novelty on JL’s repetitions. Despite starting from similar associative points, they tended to follow an independent course, perhaps fuelled by the dynamic nature of internal states. During most of the process, the work of the analyst (as it is in any analytic setting) was to listen carefully and patiently to these repetitions, and to verbalize their potential meaning to JL This often led to insight and awareness, for JL and the analyst in the moment, and the opening up of space for the introduction of new material for consideration.

## Some Brief Observations on the Generation of New Memories During the Psychotherapeutic Process

As discussed above (see Introduction), there is a growing literature suggesting that individuals with profound amnesia remain able to learn several types of new information, including forms of emotional learning. The observation of a long term psychotherapeutic process offers a unique opportunity to investigate the independence of emotional learning, for it involves the development of a new interpersonal relationship, where both new semantic and emotional information (both complex and sometimes contradictory) has to be learnt. Even though the generation of new memories during the treatment is not the main focus of this paper, we believe that it deserves brief mention.

**FIGURE 1:**
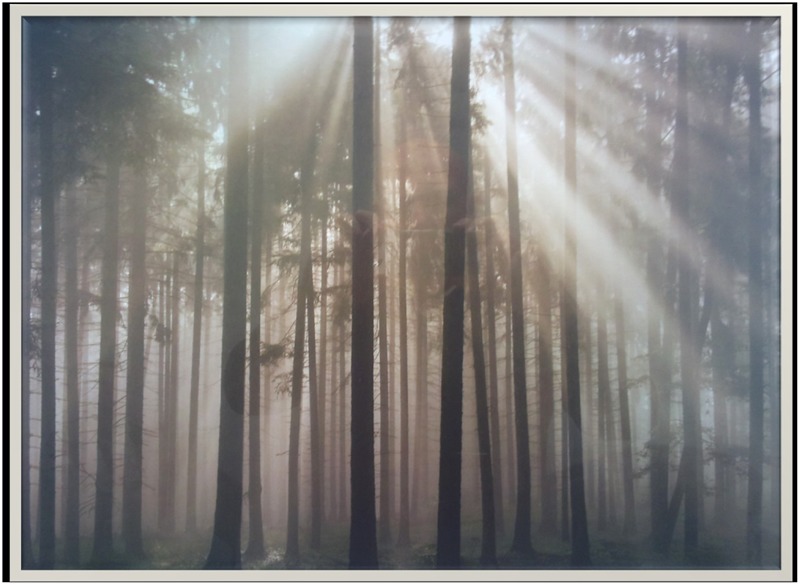
The picture of the wooden copse.

The first notable issue is that, despite his dense anterograde amnesia, JL appears to preserve *some* capacity to lay down new memories, particularly if they have an emotional nature. We have mentioned before the episode of the bridges over the river Liffey, but there are other examples. For example, there was a large photograph of a wooded copse (see **Figure 1**) on the consulting room wall, which became the source of many associative chains during the course of the treatment. To JL, this picture evoked feelings of safety, freedom, happiness and independence. It also reminded him of a location close to his original home, which he visited regularly before the brain injury. With time, this picture became quite meaningful, and JL decided to start painting it between sessions, as part of his weekly art class. To the analyst, this picture reflected some aspects of the therapeutic relationship, which JL was able to take with him, despite his inability to explicitly remember the events from the sessions. This clinical observation is interesting, in that it points to the possibility of using concrete objects to help individuals with profound amnesia in connecting to self-states that cannot be accessed via mental representations. This idea has been developed before, by Prigatano (1999), in relation to the use of drawings and paintings to help individuals with traumatic brain injury in symbolizing and elaborating their subjective experience.

There are other examples that support the idea that profoundly amnesic patients can generate new memories. For example, after 10 sessions, JL remembered the analyst’s name, and then used it consistently during each session, with no need of prompting. It appears that elements of the physical setting, where the sessions occurred, were also incorporated as new memories. On one occasion, due to the reorganization of the building, the consulting room had undergone some changes, with fixtures and fittings replaced. A large, distinctively patterned, and colorful rug, which JL would regularly contemplate during sessions, was replaced with a much smaller and more plain rug. JL noted this change almost immediately, and said: *“There’s something not quite right here, something is different about the room, I can’t quite put my finger on it.”* Unexpected separations from the analyst were also remembered by JL. For example, one session was canceled by the analyst at short notice, due to illness. In the following session, in the middle of a silence, and quite out of the blue, JL angrily confronted the analyst:

JL: So, what happened to you last week?
PM: I was sick… did you get my message?
JL: Yes
PM: What is it like not having the session last week?
JL: I don’t know I don’t know how I felt.
PM: You missed it?
JL: Yes, I did.

Given JL’s profound amnesia, it was truly surprising that he retained the absence of the analyst for an entire week. It is possible that the encoding of such an event was fuelled by strong negative emotions, perhaps linked to his history of traumatic separations. However, these observations also highlight the danger of considering memory deficits in amnesic patients as fixed, thus underestimating their capacity to be emotionally affected by interpersonal events. There is some evidence suggesting that individuals with amnestic syndromes (including dementia) preserve an ability to remember relationships (Evans-Roberts and Turnbull, 2011). The case of JL offers a novel class of evidence on the impact of interpersonal emotional *rupture* on memory encoding.

## Discussion

Patients with profound amnesia have been studied for decades. However, most of the existing evidence regarding the impact of amnesia in mental life comes from neuropsychological assessments or experimental tasks. It has been suggested by some authors that the observations of neurological patients using the analytic method can offer valuable insight into changes in emotion, motivation, and personality (Kaplan-Solms and Solms, 2000; Turnbull and Solms, 2004). This article has followed such tradition, and is the first to present data from a *long term* psychoanalytic process (72 sessions across 2 years) of an individual with profound amnesia after an anoxic episode. In relation to the aims of this paper, two main questions were explored: What is the impact of amnesia on transference and countertransference phenomena? And which technical and therapeutic modifications are required to work psychoanalytically with such amnesic individuals?

In relation to the first question, this study reported intense transference and countertransference phenomena throughout the therapeutic process, which evolved as the treatment progressed. Both transference and countertransference were importantly influenced by the amnesic deficits and by the premorbid narcissistic personality of the patient, which at times presented a sadistic quality. This combination of organic and personality elements required the implementation of important modifications to the analytic technique, such as holding the function of remembering for JL, bringing temporal continuity between and within sessions, and considering multiple levels of trauma when formulating interventions. These include premorbid trauma related to JL’s personal history, trauma associated with acquiring a brain injury, and present-day trauma associated with adjusting to life after a brain injury. Modifications to technique were also necessary at the termination phase of the treatment, in consideration of JL’s inability to remember that the treatment was coming to an end.

This case study also offers novel evidence on the neuropsychological basis of memory, particularly in relation to the dissociation of explicit and implicit memory systems. The development of a personal relationship between the analyst and JL, is consistent with data from previous studies suggesting that individuals with profound amnesia are not only able to experience emotions (Feinstein et al., 2010; Damasio et al., 2012), but also are capable of learning about the emotional valence of interpersonal relationships (Claparede, 1911/1951; Tranel and Damasio, 1990, 1993). During the course of the therapy, it was possible to observe not only how emotional valence was attached to the analyst, but also how this emotional learning process changed dynamically with the evolution of the transference. This, despite a marked difficulty in retaining and learning new information about the analyst. Nevertheless, data from this case also suggests that the inability to encode new information was not complete, since certain themes were spontaneously brought to therapy by JL on repeated occasions. These observations suggest that the generation of new memories in patients like JL is possible, but appears to be highly mediated by emotional factors. To our knowledge, this is the first case study that allows us to observe the development –and learning– of a new interpersonal relationship of this type in a patient with profound amnesia.

Our findings are of interest too for the psychoanalytic community, particularly in relation to how psychic change can take place despite a severe impairment in explicit memory, thus supporting the claims of authors who have suggested implicit memory as a core process involved in therapeutic change (see Introduction). More importantly, it confirms the relevance of implicit, or perhaps more correctly, affect-based memory systems, to transferential phenomena. Thus, this study extends the work of Turnbull et al. (2006), who offered preliminary evidence suggesting the preservation of transference phenomena in individuals with profound amnesia. However, our data is novel in that it demonstrates for the first time that transferential feelings in this population not only *unfold*, but also develop and change across the therapeutic process. This study is also novel in that it offers for the first time a description of the main features of countertransferential phenomenon when working with profoundly amnesic patients, as well as showing such information can be used to adapt the psychoanalytic tools and setting.

From a technical point of view, it is interesting to note that classic psychodynamic processes, such as hate, sadism, and narcissism, were not only observed but also addressed in the transference–countertransference phenomena. This appears to support the value of considering a relational framework with this population, particularly as regards the use of models that include the transition between part-object and whole-object forms of mental functioning.

This study has several limitations. It focuses on only one amnesic patient, with a very particular premorbid personality structure. The development of a series of cases of patients with profound amnesia would be welcomed, since it would make it possible to explore commonalities that emerge beyond the idiosyncrasies of each case. Such an approach would also contrast the influence of different analysts (including their personalities and therapeutic style), on the process. Other issues related to the setting also demand attention. It would be interesting to know whether a therapy with a higher frequency of sessions would facilitate even further the consolidation of information, or the elaboration of emotional conflicts. Similarly, since the end of this process was imposed by the analyst due to financial and logistical constraints, it would be useful to explore whether an open-ended treatment would change the dynamics of the ending phase.

This appears to be the first ever report of this nature with an amnesic patient. The investigation therefore has important theoretical implications for psychoanalytic practice, and for psychotherapy in general, and not only with regard to brain injured populations. Importantly, it raises questions concerning the mechanism of therapeutic action in psychotherapy, which has often been understood to require episodic memory. Clearly, in these instances the agent of change is not that described in many contemporary cognitive or cognitive behavioral models. Rather, change appears to be driven primarily from the affective level, and more specifically through the emotion-related components present in the analyst–patient relationship, or in the emotional field of the transference. As the transference develops, takes form and intensifies, there appears to be a correlational development and strengthening of ego function, manifested by an increased capacity to understand and tolerate loss, improved reality testing, and (arguably) improvement in depressive symptoms. Clearly, this is only an early attempt to investigate key questions – using a novel approach and emerging tools. However, we believe that it represents an interesting and important way of studying central issues in the mechanism of psychotherapy, and its neuroscientific basis.

## Ethics Statement

This study was carried out in accordance with the recommendations of the following organizations: Headway Ireland (Acquired Brain Injury Service) and Bangor University, North Wales.

## Author Contributions

PM: Lead author, clinician, and clinical research. CS: Co-author and collaborator, facilitating clinical and theoretical discussion of topic and material. SD: Organization liaison person, facilitation, clinical supervision, manuscript feedback, neuropsychological assessment of participant and interpretation of same. OT: Ph.D. supervisor to lead author (PM), research design, manuscript supervision, and editorial feedback.

## Conflict of Interest Statement

The authors declare that the research was conducted in the absence of any commercial or financial relationships that could be construed as a potential conflict of interest.

## Footnotes

1 In order to protect JL’s anonymity, names and places have been altered. Informed consent was obtained from JL to publish anonymized findings.

2 Craic – colloquial Irish term for fun, however, in this instance it can be understood as “all that sort of thing.”

3 A unique reference number that helps patients access social welfare benefits, public services and information.
